# In-situ investigation of the decomposition process in cold-rolled Nb53Ti47 alloy

**DOI:** 10.1016/j.heliyon.2024.e26543

**Published:** 2024-02-16

**Authors:** Erzsebet Nagy, Ferenc Kristaly, Viktor Karpati, Valeria Mertinger

**Affiliations:** a HUN-REN-ME Materials Science Research Group, Institute of Physical Metallurgy, Metalforming and Nanotechnology, University of Miskolc, H-3515, Miskolc-Egyetemvaros, Hungary; b Institute of Mineralogy and Geology, University of Miskolc, H-3515, Miskolc-Egyetemvaros, Hungary; c Institute of Physical Metallurgy, Metalforming and Nanotechnology, University of Miskolc, H-3515, Miskolc-Egyetemvaros, Hungary; d Wigner Research Centre of Physics, H-1121, Budapest Konkoly Thege Miklos 29-33., Hungary

**Keywords:** NbTi alloy, Spinodal decomposition, α-Ti precipitation, In-situ XRD, High-temperature XRD

## Abstract

The multi-layer composite development primarily aims to develop and test the components of the next generation of hadron colliders (e.g., Large Hadron Collider - LHC) consisting of superconducting raw materials. Multilayer sheet is very similar to the commonly used NbTi wire products, a 2D version of the commercial wire. These composites consist of layers such as NbTi superconductor, Nb diffusion barrier (between NbTi and Cu) and Cu stabilizer. In β-NbTi superconducting alloys, α-Ti precipitates are primary flux pinning centers that maintain stable superconductivity. A multi-step series of heat treatments and cold-forming processes can develop the flux pinning centers. Practically, this process means three heat treatments of constant period and temperature and drawing or rolling between the heat treatments.

The study aimed to describe the behavior of the cold-rolled (ε = 3.35) Nb53Ti47w% alloys during isothermal heating at 673 K as a function of heating time. The processes during the aging were investigated by the in-situ XRD method in the heating chamber. The X-ray diffraction patterns were evaluated by Rietveld refinement. The thermally activated spinodal decomposition and precipitation processes were described based on the phases identified at the individual heat treatment steps and their lattice parameters. The in-situ study also revealed an increase in α-Ti precipitation with time and decomposition that co-occurs. This is the basic study that prepares the applicability of the alloy.

## Highlights

• In-situ X-ray analysis using a single sample, as opposed to heat treatment of different individual samples as a function of time.
• During the in-situ heat treatment, the processes can be monitored by taking diffractograms at pre-determined steps.
• Developing the hexagonal α-Ti in the in-situ heated sample after the decomposition of the β-NbTi solid solution.
• The Rietveld refinement revealed not only changes in the amount of developing phases but in their crystallographic data too.

## 1 Introduction

The widely used NbTi superconductor materials are present in MRI equipment, particle accelerators, and many more magnetic shielding applications. The NbTi alloys with a Ti-content of 45–50 wt% are characterized by high strength and formability with a high critical current density (Jc) which drops to zero, below the electrical resistance of a superconducting material.

Under the influence of the thermomechanical treatment, α-Ti precipitates develop in the β phase in the NbTi alloys [[1], [2], [3]]. The number of precipitates increases by increasing the heat treatment temperature; The effect is similar if the period of heat treatments increases at a constant temperature. The value of Jc increases linearly by increasing the volume fraction of α-Ti precipitates [4,5[6], [7], [8]].

**Fig. 1:**
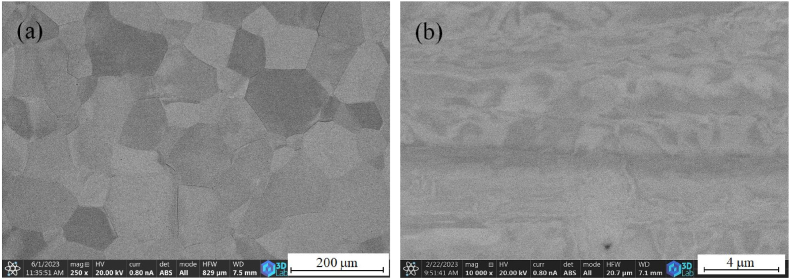
Microstructure on the cross-section of the samples. (a) original hot-rolled state; (b) initial cold-rolled state.

β-isomorphous Ti-alloys have a high degree of metastability. Depending on the β-stabilizer content, the supersaturated solid solution may decompose into the equilibrium α and β phases. In TiNb β-isomorphous systems (Fig. 1), in the region referred to as a "miscibility gap", a homogeneous, single-phase β0-solid solution decomposes into a thermodynamically stable mixture of two bcc phases, one Ti-rich (β1) and the Nb-rich (β2) (1). Then β1 transforms into α and β2 phase in a monothetic reaction (2) [9,10].

(1) β0→β1+ β2

(2) β1→α+β2

The spinodal decomposition phenomenon [11,12] can be observed in different types of alloys, e.g., HEA [[13], [14], [15], [16]], FeCr alloy [17], Mn–Cu alloys [18,19], Cu-based alloys [20], Mo–V alloys [21], U–Nb alloy [22], FeMnAlC [23], InAlN alloy [24].

In the Ti-X system, Flower et al. [25] earlier established in Ti-4 wt% Mo alloys a spinodal decomposition reaction, which is conditional upon the formation of the orthorhombic α" form of martensite. A detailed study has been made of the precipitation reactions that occur when the Ti–Mo system's martensites are aged. Moffat et al. [26] summarized the calculation existence of β-phase decomposition in the metastable phase diagram of the Ti–Nb and Ti–V, Ti–Mo system. Zhang et al. [27], the thermodynamic properties of the Nb–Ti system have been calculated for the equilibrium and the metastable phases. Wang et al. stable and metastable phase equilibria, the metastable chemical spinodal curve of β phase, and thermodynamic properties are simulated with the optimized parameters [28]. A phase field method was applied to simulate the spinodal decomposition in Zr–Nb alloys analogous to the Ti–Nb system. The spinodal region on the Zr– Nb phase diagram was calculated by the phase field method by considering the interfacial energy and elastic strain energy contribution to the total Gibbs free energy. There have been many research outcomes on the typically superconducting alloys with a 45–50 wt% Ti content. Boenisch et al. [29] investigated the thermal stability of NbTi alloys in a wide concentration range. Moon et al. [30] and Kim et al. [31] showed the relationship between the initial structure and the critical current density, while other authors observed changes in the microstructure due to various external influences [2,6,[32], [33], [34]]. Storozhilov et al. [35] investigated the effect of severe plastic deformation on the decomposition of supersaturated solid solution in Nb–48.5 wt % Ti. It has been established that an increase in the true plastic prestrain of the NT-50 alloy from 4 to 7.6 leads to the significant acceleration of supersaturated solid solution decomposition at all treatment times (from 50 to 2000 h). In Ti–Nb alloys, certain non-metallic alloying elements affect the course of spinodal decomposition, such as oxygen and nitrogen. Using the phase-field method, Ishiguro et al. [36] investigated the β-phase spinodal decomposition in oxygen-added Ti–Nb alloys simulated at 700–1073 K. The results show that adding low amounts of oxygen remarkably enhances the β-phase spinodal decomposition at high temperatures. The volume fraction and composition of the β1 and β2 phases depend on the heat treatment condition, alloy composition, and oxygen content. The equilibrium niobium composition of the β1 phase slightly decreases as oxygen composition increases. On the other hand, the equilibrium niobium composition of the β2 phase remarkably increases as oxygen composition increases. Kobayashi et al. [37] found that adding oxygen or nitrogen to Ti–10V alloy increases the wavelength of the modulated structure by spinodal decomposition. The hardness's absolute value increased as the alloys' oxygen or nitrogen content increased.

This study investigated the decomposition and precipitation processes in a cold-formed Nb47w%Ti alloy by heat treatment with an in-situ X-ray diffraction method. In the case of the NbTi alloy deformed to the extent of ε = 3.35, a heat treatment procedure was performed at 673 K for different periods using the in-situ investigation method. SEM was used to investigate the microstructure of the in-situ heat-treated sample, and the diffractogram series made during the XRD investigation was evaluated by Rietveld refinement.

## 2 Materials and methods

### 2.1 Material

A high-purity superconductor alloy composed of 53w%Nb and 47w%Ti was used for the investigations (Table 1.).

**Table 1:** Composition of experimental Nb53Ti47 alloys [w%].

Ti	C	O	Cr	Fe	Al	N	H	Ta	Si	Ni	Cu	Nb
47.35	0.005	0.038	0.004	0.005	0.005	0.0074	0.001	0.0054	0.005	0.003	0.003	bal.

The cold forming of the alloy was performed on a von Roll experimental rolling stand. During the cold rolling, the alloy was rolled to a final thickness of 0.4 mm from the original thickness of 20 mm in multiple reversal punctures at 30 m/min speed. Samples of 4 × 4 mm were cut from the cold-rolled sheet. The logarithmic deformation of sheet ε = 3.35, where ε = ln(H_0_/H_1_) H_0_ initial plate thickness, H_1_ current cold-rolled plate thickness. The small cold-rolled plate was cleaned and degreased in ethanol and subjected to heat treatment by in-situ methods.

### 2.2 High temperature XRD

In-situ heat treatments were carried out in the HTK 1200 N heating chamber part of the Bruker D8 Discover XRD system. In the experiment, the sample was heated with a 60K/min heating rate to 673 K, followed by isothermal treatment under vacuum, and after 0; 5; 10; and 20 h, diffractograms were recorded in the 33–98°(2θ) range. The scan time at each step was about 31 min. The sample was then allowed to cool with a 60K/min cooling rate. The in-situ study aimed to observe more precisely the isotherm kinetics of decomposition and α-Ti precipitation.

The phase analyses of the in-situ heat-treated sample were done by Bruker D8 Discover XRD equipment with Cu Kα(1,2) source (40 kV and 40 mA generator settings). Parallel beam geometry with a Göbel mirror was applied in a range of 33–98°(2θ), an increment of 0.007°(2θ), and a counting time of 0.40 s by using a LynxEye X-ET energy dispersive detector in 0D mode high energy resolution. The evaluation was performed in Bruker EVA 5.0 software, a PDF2 database for identifying phases, and Bruker TOPAS 6.0 software for Rietveld refinement. The instrument is aligned with SRM NIST 1976b corundum standard and calibrated with the SRM NIST 640d silicon powder standard. In the course of Rietveld refinement, the measured curve obtained from the sample is fitted with the cumulative calculated curve obtained from the single curves calculated from the identified phase’s crystal structures, comprising all the corrections (PO) and broadening modeling (size and strain) that we apply. We aim to minimize the difference between the measured and calculated curves. Conclusions concerning the phenomena (e.g., orientation) causing the difference can be drawn from the shape of the difference curve and from the absolute value of residual peaks on the difference curve. After the identification of phases, the calculated patterns are fitted to the measured diffractogram without any PO correction, theoretical unit cell parameters, peak broadening modeled with crystallite size calculation. Since unit cell parameters reflect the presence of alloying elements in the phases, this is the first parameter to be fitted. The following step requires performing the PO correction to the (hkl) peaks with the March-Dollase function we can correct for two (hkl) series – defined by the user – of each phase. The presence of lattice strain is reasonable in the case of an orientated structure obtained by thermal or mechanical processing. The last refinement procedure is completed by fitting the Debye-Waller thermal parameters.

### 2.3 Microstructure analysis

The heat-treated sample was prepared for the microstructure investigation. The plate-shaped sample built in resin was ground on 320# SiC paper, then polished with MD Largo with 9 mm Largo suspension for 5 min, then OPS non-dry emulsion for 25 min with Struers Tegramin20 equipment. The preparation was finished by etching with a solution of nitric acid, hydrogen fluoride, and water for 30 s. The sample' microstructure was investigated using a Thermo Scientific Helios G4 Plasma Focused Ion Beam Scanning Electron Microscopy (PFIB SEM). High-resolution elemental EDS maps were created to visualize the distribution of the constituent elements in the specimen by two-dimensionally displaying the characteristic X-ray intensities or the concentrations of the elements.

## 3 Results and discussion

The microstructure of the hot rolled state base material and the cold-rolled sample (ε = 3.35) is shown in Fig. 1(a,b). The initial structure consists of a supersaturated, body-centered cubic β-NbTi phase with slip lines to accommodate a high degree of deformation. It is also clear from the cross-sectional image that the structure is abnormally banded, indicating the inhomogeneity of the solid solution, which resulted in non-uniform deformability during rolling. The banding persists in the order of mm along the length of the ribbon.

### 3.1 Evolution of phases by heat treatment

**Fig. 2:**
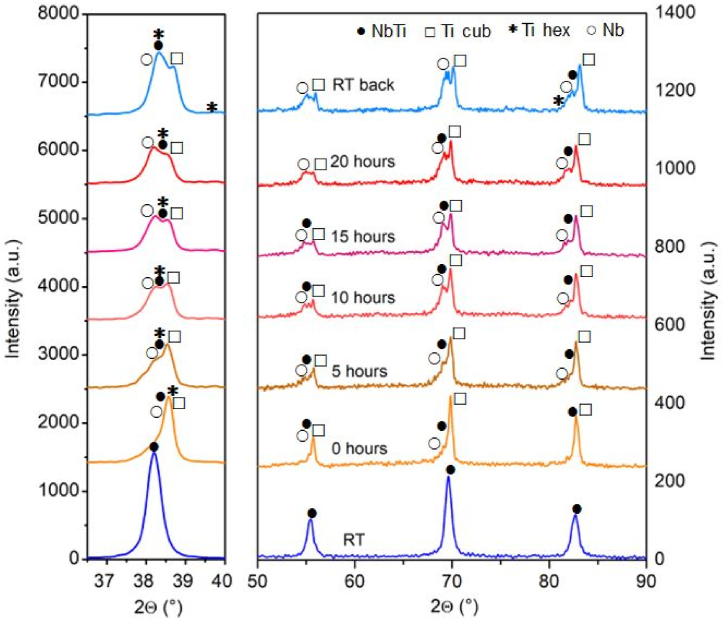
Diffractograms were recorded during the in-situ investigation, when the sample was heated at up to 673 K, followed by isothermal treatment under vacuum, and after 0; 5; 10; and 20 h, then cooled back RT. (a) 36–40°(2θ) range; (b) 50–90°(2θ) range.

During the in-situ heat treatment, the processes can be monitored by taking diffractograms at pre-determined steps utilizing the in-situ investigation method. The in-situ investigation was performed for 20 h based on the results of the furnace heat-treated samples. Following the heat treatment, body-centered cubic Nb rich (labeled as Nb, space group: Im-3m), body-centered cubic Ti (labeled as β-Ti space group: Im-3m), hexagonal Ti (labeled as α-Ti space group: P63/mcm) phases appear in addition to the body-centered cubic β-NbTi phase (space group: Im-3m). The diffractograms determined during the in-situ investigation can be seen in Fig. 2(a,b).

**Fig. 3:**
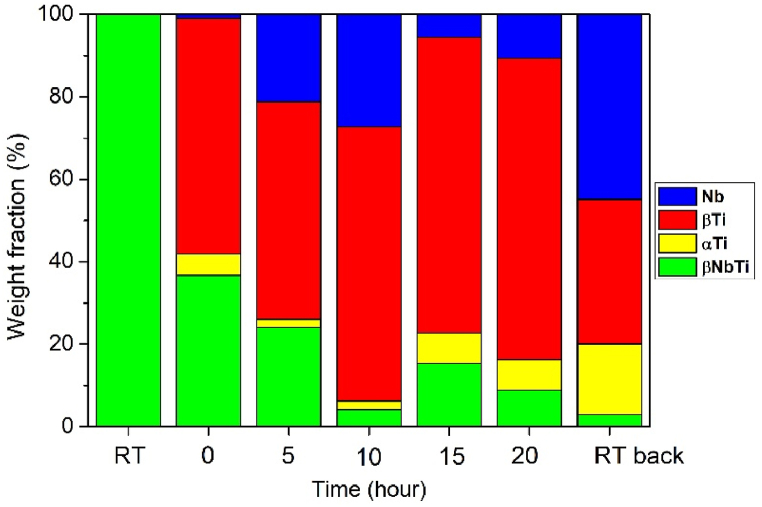
The weight percent of in-situ heat-treated sample as a function of heat-treating time [w%].

The diffractogram taken at room temperature (RT sample) at the beginning of the investigation shows the initial cold-rolled condition (ε = 3.35). The second diffractogram was taken when the sample reached 673 K (0 h). Approximately 11 w% of non-stoichiometric reaction products crystallize; based on database identification, mainly TiO_0.32_ and TiN_0.3_ formation could be identified. However, the phenomena could be due to the imperfect vacuum due to the dissolved oxygen and nitrogen rather than the oxide and nitride nature of the products, it’s more likely that elements contained in the rolled metal are fixed in these structures. This assumption is also supported by the identical space group and symmetry of TiO_0.32_ and TiN_0.3_ to that of α-Ti. These phases appearing during the in-situ test could be formed as a thin layer on the sample’s surface as a narrow upper part of the X-ray beam irradiated sample. The structure and stable percentage of oxygen-containing phases contradict Ti oxidation in the air due to vacuum contamination since that could result in TiO_2_ formation. The amount of phases determined by Rietveld refinement for the in-situ heat-treated sample is in Table 2. The trends in the function of aging time are shown in Fig. 3. The application of the Rietveld refinement in the case of oriented structured material is described in Ref. 38.

**Table 2:** The weight percent of the in-situ heat-treated sample in the different heat treatment steps (w%, error five relative percent).

Sample	β-NbTi	α-Ti	β-Ti	Nb	Oxides
RT	100	0	0	0	0
0-h	33	5	51	1	11
5-h	21	2	47	19	11
10-h	4	2	58	24	13
15-h	13	6	62	5	13
20-h	8	6	64	9	13
RT back	3	15	31	40	12

### 3.2 Transformation processes during heat treatment

**Fig. 4:**
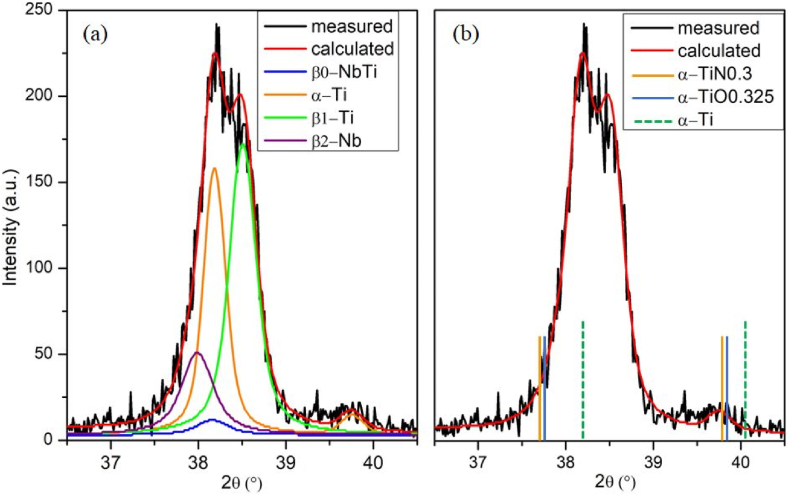
Decomposition of β-NbTi by Rietveld refinement (20-h state) (a), The peak position of different α-Ti phases (b).

Based on the diffractograms, the decomposition and precipitation processes start when the temperature reaches 673 K. During the heat treatment process, the supersaturated β-NbTi solid solution becomes thermodynamically unstable. It spontaneously decomposes in two phases (Nb, β-Ti) without nucleation. The spontaneous decomposition of the unstable phase into stable phases is irreversible. The development of the shoulder on reflection (2Θ = 38.51°) due to decomposition and the change of peak shape can be observed on the diffractograms (Fig. 4(a)). The appearance of an α-Ti reflection shows that the precipitation process also occurs (Fig. 4(b)). The presence of α-Ti reflections in the diffractogram taken at each 5th hour for 20 h proves the progression of the precipitation process. The equilibrium phases developing because of spinodal decomposition will remain in the microstructure even after being cooled back to room temperature (RT back state). However, it is important to emphasize that the Rietveld refinement method is performed for all reflections over the full angle range in all cases. The amount of Nb and β-Ti, the products of spinodal decomposition, increase steadily at the expense of the initial β-NbTi. The increase of α-Ti is clear from 10h in the studied time interval.

**Fig. 5:**
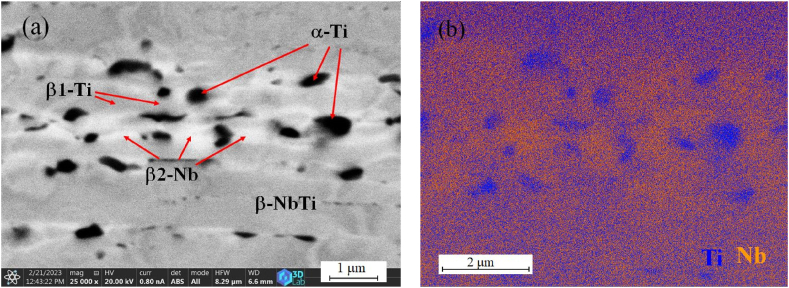
SEM image and EDS map on the same area of the cross-section of the in-situ heat-treated sample. (a) SEM image of the surface; (b) Nb and Ti element on the surface by EDS Map (Nb - orange, Ti - blue). (For interpretation of the references to colour in this figure legend, the reader is referred to the Web version of this article.)

The results of the SEM investigation on the cross-section of the heat-treated sample are shown in Fig. 5. The dark spots in the microstructure in Fig. 5(a) are the high Ti content phase according to the EDS map. In this area, Nb is not detectable. The matrix contains Ti and Nb with an inhomogeneous distribution. The colour differences between darker and lighter areas refer to the concentration of certain elements. The significantly orange area in the matrix shows a higher Nb content, while the bluer areas are enriched in the Ti element (Fig. 5(b)). In the microstructure, the light areas around the dark α-Ti precipitates are depleted in titanium due to the precipitation process, which are the orange parts visible in the vicinity of the blue (Ti) precipitation on the EDS map. In the less characteristic orange areas, the Nb/Ti ratio moves less towards Nb.

### 3.3 Interpretation of crystallographic parameters

**Fig. 6:**
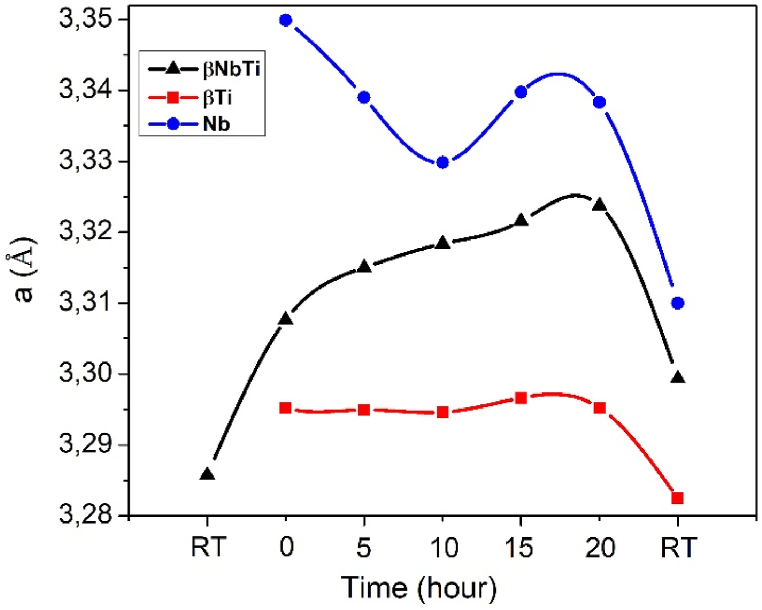
Change in the lattice parameters of body-centered cubic phases during the in-situ investigation. Black line: β-NbTi; red line: β-Ti phase; blue line: Nb phase. (For interpretation of the references to colour in this figure legend, the reader is referred to the Web version of this article.)

The Rietveld refinement revealed not only changes in the amount of phases but also changes in their crystallographic data. The variation of the lattice parameters of the cubic lattice phases is shown in Fig. 6.

The decomposition of the β-Nb47Ti53 phase for a Ti-rich (β1) and an Nb-rich (β2) phase starts during the first step of heating. At the same time, the precipitation process when the Ti-rich β1 participates α and β2 phase in a monotectoid reaction also begins. During the decomposition of β-NbTi, the same type of bcc phases develops, which differ merely as far as the atoms occupying the lattice points are concerned. As the atomic radii of Nb and Ti elements have similar sizes, they can easily substitute each other in the crystal lattice. The lattice parameter of phases developing because of spinodal decomposition: the β1 phase (3.299 Å) is smaller, while the β2 phase (3.310 Å) is higher compared to the initial β-NbTi phase (3.285 Å), which increases according to its thermal dilatation. The value of *a* lattice parameter increases in the case of β-NbTi and β-Ti phases by increasing the holding time. The lattice parameter of Nb really decreases in the first stage of heat treatment (Fig. 6). The lattice parameter of the bcc phases increases due to the solution of the alloying element and the thermal dilatation. The β-phases developing under the influence of decomposition are never pure phases but contain dissolved alloying elements (practically, the β1 one contains dissolved Nb atoms while the β2 contains dissolved Ti atoms). The change in lattice parameters shows a similar tendency in all three bcc phases. The decreased lattice parameters measured at room temperature result from eliminating thermal dilatation. The increased lattice parameter developed after cooling is not due to the dissolved alloying element in the Nb phase; since the Ti atom dissolving in the Nb lattice should reduce the value, practically a value smaller than the theoretical Nb value should be obtained. In the case of the Nb phase, the increased lattice parameter value is caused by the dislocations present due to the large plastic deformation (ε = 3.35).

Developing the hexagonal α-Ti in the in-situ heated sample after the decomposition of the β-NbTi solid solution begins at the expense of the Ti-rich phase. The values of *a* and *c* lattice parameters increase owing to the thermal dilatation of the lattice as well as the dissolving of solved atoms into the lattice as the heating time increases. During the qualitative analysis of the in-situ heated sample, we observed that the theoretical peaks of the pure α-Ti phase do not match the measured reflections (Fig. 4(b)). However, it showed a good match for the two other phases. These phases have the same space group, representing non-stochiometric oxide and nitride compositions with a somewhat larger unit cell (Table 3.). Wriedt et al. stated that α-Ti could dissolve its alloying element in the Ti–N system [39]. For the O–Ti system, Okama et al. found that α-Ti can dissolve 0-33.3a% of oxygen [40]. Practically, this means that for a given series of planes (hkl), the phase with a larger lattice parameter (a, c) will have a larger lattice plane distance (d), and thus, the reflection obtained from the plane will be shifted towards a smaller 2Θ angle value.

**Table 3:** The unit cell parameters and main peaks of α-Ti and related phases (Ref. PDF2 database).

Phase	System	Space group	a(Å)	c (Å)	(101)	
					d_(101)_ (Å)	2Θ_(101)_ (°)
α-Ti	hex	P63/mmc (194)	2.9505	4.6826	2.243	40.170
α-TiN_0.3_	hex	P63/mmc (194)	2.9737	4.7917	2.268	39.709
TiO_0.3_	hex	P63/mmc (194)	2.9700	4.7751	2.264	39.771

After the decomposition of β-NbTi during in situ heating, as soon as the α-Ti precipitation process begins, the developed α-Ti phase shows an increasing lattice parameter due to the oxygen and nitrogen uptake facilitated by the thermal dilatation of the lattice (Table 4.). The decreased lattice parameter in the “RT back” state after the cessation of thermal expansion is caused by shrinking lattices when the reduction of crystallite volume also leads to the decrease of interplanar distances. Thus, the deconvolution of α-Ti and non-stoichiometric phases becomes less precise. Due to the overlapping of the broadened peaks, the calculated lattice parameters are at least partly a mixed value of α-Ti and non-stoichiometric phases.

**Table 4:** The changing of the lattice parameters of α-Ti during in-situ heating.

	α-Ti	
	a (Å)	c (Å)
RT	–	–
0-h	2.9180	4.7015
5-h	2.9786	4.7057
10- hours	2.9813	4.7150
15-h	2.9767	4.7209
20-h	2.9769	4.6976
RT back	2.9729	4.6958

## 4 Conclusions

The in-situ X-ray study of a cold-rolled Nb47w%Ti alloy revealed the spinodal decomposition of β-NbTi and the precipitation of α-Ti. The developed microstructure was investigated, and the amounts of developed phases were determined by Rietveld refinement as a function of heating time at 673 K. The progress of the decomposition and precipitation processes was followed by an in-situ X-ray diffraction method by detecting the changes in the sample at determined time intervals. The results are summarized as follows:

• Under isothermal conditions, the metastable β-NbTi phase is almost completely decomposed as the heat treatment time progresses, only a few percent remain in the sample at the end of the heat treatment. The amount of β1 phase increase with the heat treatment time, and the amount of solid solution β2 does not change significantly.
• As a result of the monotectic transformation of β1, the weight percent of the appearing α-Ti and β2 phases increase. The amount of α-Ti is doubled, while the amount of β2 increases four times.
• After re-cooling, the spinodal decomposition of β-NbTi is completed, and with the transformation of the β1 solid solution, the amount of α-Ti precipitates and the β2 phase increases.
• In the microstructure, areas with different compositions can be observed in the images created with backscattered electrons; these areas with various grey levels correspond to solid solutions with different compositions formed during spinodal decomposition.
• The presence of solid solutions in multiple β-phases is supported by the lattice parameters obtained by Rietveld refinement, also demonstrating the presence of various alloying element ratios in the nominally pure phases.

A further important finding of the study is that it highlights the advantages of in-situ X-ray analysis using a single sample, as opposed to the uncertainty caused by heat treatment of different individual samples as a function of time.

The heat treatment experiment of the Nb53Ti47 alloy provided essential information for the development of the multilayer composite. Getting to know the processes that take place during the in-situ examination contributed to the optimization of the production process of the composite consisting of a combination of plastic deformation and heat treatment, especially regarding the formation of a-Ti precipitates, which provide the pinning centers in the magnetic shielding material.

## Funding

This work was supported by the National Research, Development and Innovation Fund (ÚNKP-20-5 New National Excellence Program of the 10.13039/501100015498Ministry for Innovation and Technology, Doctoral Student Scholarship Program of the Co-operative Doctoral Program of the Ministry of Innovation and Technology); 10.13039/501100003825Hungarian Academy of Sciences (János Bolyai Research Scholarship); and 10.13039/501100018818National Research, Development and Innovation Office (Grant Contract reg. nr.: TKP-17-1/PALY-2020).

## Data availability statement

The datasets used and analysed during the current study available from the corresponding author on reasonable request.

## CRediT authorship contribution statement

**Erzsebet Nagy:** Writing – review & editing, Writing – original draft, Investigation, Conceptualization. **Ferenc Kristaly:** Writing – review & editing, Validation, Methodology, Data curation. **Viktor Karpati:** Visualization. **Valeria Mertinger:** Writing – review & editing, Supervision.

## Declaration of competing interest

The authors declare the following financial interests/personal relationships which may be considered as potential competing interests: Erzsebet Nagy reports financial support was provided by 10.13039/501100003825Hungarian Academy of Sciences. Viktor Karpati reports financial support was provided by 10.13039/501100015498Ministry for Innovation and Technology Hungary. Erzsebet Nagy reports financial support was provided by 10.13039/501100015498Ministry for Innovation and Technology Hungary. Valeria Mertinger reports a relationship with 10.13039/100012470CERN that includes: non-financial support. Non financial cooperation agreement between the University of Miskolc and CERN (2019) for the development and testing of components of the Large Hadron Collider - LHC, consisting of superconducting materials. Non financial cooperation between the Institute of Physical Metallurgy, Metalforming and Nanotechnology of the University of Miskolc and the MTA Wigner Physics Research Center on the development of functional multi-layer composite structures.
